# Research on the Dynamic Feedback Mechanism of Fiscal Policy Regulation Under COVID-19: Evidence From China

**DOI:** 10.3389/fpubh.2022.931135

**Published:** 2022-07-05

**Authors:** Shu Wang, Henan Gao, Baicheng Zhou

**Affiliations:** ^1^School of Economics, Jilin University, Changchun, China; ^2^School of Business, Changchun Guanghua University, Changchun, China

**Keywords:** fiscal policy, dynamic feedback, rule, discretionary, China, COVID-19

## Abstract

The repeated outbreak of COVID-19 epidemic has brought a heavy blow to the world economy. Fiscal policy is one of the important macro-control measures to pull the economy out of the quagmire, and it is necessary to study the implementation of fiscal policy under the epidemic. Due to the relatively abundant resources of the Chinese government, this study uses China as the research object to study the orientation of fiscal policy under COVID-19 epidemic. We use fiscal policies and a large amount of macroeconomic data to identify fiscal policy and macroeconomic regulation's dynamic mechanism in China. Our findings indicate a dynamic feedback relationship between expenditure-based and revenue-based fiscal policy tools, output gaps, and deficit scales. Before the global economic crisis, fiscal policy can play a good role in adversely regulating the economy, and the difficulty of adjustment after the crisis has increased significantly. During COVID-19 epidemic, the interaction time between variables related to fiscal policy increased, suggesting that the implementation of fiscal policy during the epidemic should be particularly cautious.

## Introduction

The COVID-19 epidemic has swept the world since the end of 2019, bringing a heavy blow to the economies of various countries (1). In order to deal with the economic downturn caused by the COVID-19 epidemic and prevent secondary disasters caused by the COVID-19 epidemic as much as possible, the government has to rely on the role of macro-control to intervene. It often takes a while for monetary policy to act on the macro economy, and it is difficult to respond to a sudden negative shock like the COVID-19 outbreak in a timely manner. In contrast, the effect of fiscal policy is more immediate. When the economy is severely negatively impacted, studying the dynamic feedback mechanism of fiscal policy on the macroeconomy is conducive to making marginal contributions to research in related fields and conducive to in-depth understanding of fiscal policy regulation. It has important reference value for the formation of a fiscal system that matches the modernization of national governance. Fiscal policy is an essential pillar of national governance, and the level of fiscal governance is related to the country's stability and development. The Chinese government has many resources and has a relatively strong ability to influence economic sectors (2, 3). Therefore, Chinese data is selected as the research object of this paper to study how fiscal policy is adjusted when the economy suffers a negative blow. However, the problem is that the implementation of fiscal policy is not consistent over a long time. The past handling methods may be seriously divorced from the actual economic situation. Therefore, exploring the implementation preferences of China's fiscal policy should start from a dynamic perspective, while considering many economic variables that may be involved in the process of fiscal policy implementation, so as to make the empirical results fit the actual economy.

This paper constructs a non-linearity reflecting the dynamic and time-varying relationships among fiscal policy tools, output gaps, and fiscal deficits. The model analyzes the dynamic feedback mechanism of fiscal policy and macroeconomics. The main contribution is the practical analysis of the dynamic adjustment mechanism between fiscal policy tools, output gap, and fiscal deficit scale, which can be regarded as an essential supplement to current fiscal policy empirical research. It is of great significance to understand the operational characteristics of the government's fiscal policy during the crisis.

The rest of the paper is organized as follows. The second part is a literature review. The third part introduces the relevant theory of fiscal policy rules and proposes the theoretical framework used in this paper. The third part introduces the empirical analysis method. The fourth part introduces the empirical analysis method. The fifth part is data introduction and selection. The sixth part is the empirical results. The last part is the conclusion.

## Literature Review

The research on the effectiveness of fiscal policy has become a key research issue in the field of macro-control in various countries, and it is primarily reflected in the analysis of the policy effectiveness of fiscal rules under a dynamic stochastic general equilibrium system (4, 5). In the discussion on the effectiveness of fiscal policy, the question of the mode of policy operation cannot be avoided, that is, should the government insist on using fiscal policy rules that are pegged to certain macroeconomic variables in the face of a severe negative blow to the economy such as the COVID-19 pandemic, or should the government use discretionary flexibility that adjusts to economic circumstances? On this issue, the debate over policy rules versus discretion has a long history. Keynes, Samuelson and others believed that camera decision-making was more effective, and “Keynesianism” was highly sought after during the Great Depression (6, 7). However, Friedman, Kydland and others took a cautious objection (8–10), arguing that it will lead to problems such as dynamic inconsistency and high welfare costs, which itself will become one of the causes of economic cycles (11–13). From the perspective of the characteristics of macroeconomic regulation, policy rules and discretion have their own advantages and disadvantages (14, 15). Therefore, the expanded policy rules that are partially unified and respond synchronously with changes in other economic variables have been favored by scholars (16–19). The research on how China's finance is regulated has been concentrated in recent years (20–24), the existence and effect of rules have been estimated.

Combing through the literature in the past can reveal that although the research has achieved many meaningful conclusions in many aspects, the orientation of fiscal policy when the economy has suffered a severe negative blow has not been thoroughly explored. This paper focuses on the outbreak of the COVID-19 epidemic, which has caused a huge blow to the economic system, and studies the dynamic characteristics and implementation orientation of China's fiscal policy, aiming to provide experience and reference for other countries' fiscal policy implementation in special times.

## Theoretical Analysis

The government's expenditure-based and revenue-based policy tools will be adjusted according to the actual government debt scale and the output gap (25, 26).


(1) $$\begin{array}{r} {Ĝ}_{t}^{exp}=\eta_{G^{exp}}{Ĝ}_{t\text{-}1}^{exp}+\lambda_{G^{exp}y}{ŷ}_{t-1}+\lambda_{G^{exp}b}{\hat{D}}_{t-1} \end{array}$$

(2) $$\begin{array}{r} {Ĝ}_{t}^{rev}=\eta_{G^{rev}}{Ĝ}_{t\text{-}1}^{rev}+\lambda_{G^{rev}y}{ŷ}_{t-1}+\lambda_{G^{rev}b}{\hat{D}}_{t-1} \end{array}$$

Simultaneously, considering the lack of data on debt balance in China, fiscal deficits are more effective in restraining government behavior and easier to observe than debt balances (22). Moreover, the discretionary choice has often become the main policy tool of the fiscal sector, which means that fiscal policy is not a completely regular form, so the fiscal policy model is described in detail as:


(3) $$\begin{array}{r} {Ĝ}_{t}^{\exp}=\eta_{G^{\exp}}{Ĝ}_{t\text{-}1}^{\exp}+\lambda_{G^{\exp}y}{ŷ}_{t-1}+\lambda_{G^{\exp}b}{\hat{D}}_{t-1}+e_{G^{\exp},t} \end{array}$$

(4) $$\begin{array}{r} {Ĝ}_{t}^{rev}=\eta_{G^{rev}}{Ĝ}_{t-1}^{rev}+\lambda_{G^{rev}y}{ŷ}_{t-1}+\lambda_{G^{rev}b}{\hat{D}}_{t-1}+e_{G^{rev},t} \end{array}$$

Among them ${Ĝ}_{t}^{\exp}$ and ${Ĝ}_{t}^{rev}$ are the proxy variables of expenditure and revenue-based fiscal policy tools. ŷ and $\hat{D}$ are the output gap and the government deficit's size. $\eta_{G^{\exp}}$, $\eta_{G^{rev}}$ depicts the smoothness of fiscal policy operations. That is, current government expenditures and revenues will be pegged to the previous government to a certain extent. $e_{G^{\exp},t}$, $e_{G^{rev},t}$ respectively represent the unobservable parts of government expenditure and government revenue in fiscal policy tools, reflecting the discretionary degree of fiscal policy tools. $\eta_{G^{\exp}}{Ĝ}_{t\text{-}1}^{\exp}\text{+}\lambda_{G^{\exp}y}{ŷ}_{t-1}+\lambda_{G^{\exp}b}{\hat{D}}_{t-1}$, $\eta_{G^{rev}}{Ĝ}_{t\text{-}1}^{rev}\text{+}\lambda_{G^{rev}y}{ŷ}_{t-1}+\lambda_{G^{rev}b}{\hat{D}}_{t-1}$ respectively mean the regulatory changes in fiscal policy instruments.

However, just as the empirical evidence of the non-linear form of Taylor rule, we also believe that the target of fiscal policy in China is not consistent in the long term, and potential targets time-varying. Combined with the theoretical viewpoints above, we further characterize the fiscal policy model as:


(5) $$\begin{array}{r} {Ĝ}_{t}^{\exp}=\eta_{G^{\exp},t}{Ĝ}_{t\text{-}1}^{\exp}+\lambda_{G^{\exp}y,t}{ŷ}_{t-1}^{*}+\lambda_{G^{\exp}b,t}{\hat{D}}_{t-1}^{\text{*}}+e_{G^{\exp},t} \end{array}$$

(6) $$\begin{array}{r} {Ĝ}_{t}^{rev}=\eta_{G^{rev},t}{Ĝ}_{t\text{-}1}^{rev}+\lambda_{G^{rev}y,t}{ŷ}_{t-1}^{*}+\lambda_{G^{rev}b,t}{\hat{D}}_{t-1}^{\text{*}}\text{+}e_{G^{rev},t} \end{array}$$

${y^{*}}_{t-1}$ represents the deviation of output from the potential time-varying production and ${\hat{D}}_{t-1}^{\text{*}}$ represents the fiscal deficit's deviation from the time-varying fiscal deficit target. There should be a “countercyclical” effect between the output gap and deficit changes and the government's fiscal policy in an ideal state (22, 27). This paper also adds factor enhancement ideas in the empirical research part's fitting process to avoid loss variables distorting the results (28–30), we characterize the fiscal policy model as:


(7) $$\begin{array}{l} {\hat{G}}_{t}^{\exp}\;=\;\eta_{G^{\exp},t}{\hat{G}}_{t-1}^{\exp}+\lambda_{G^{\exp}y,t}{\hat{y}}_{t-1}^{*}+\lambda_{G^{\exp}b,t}{\hat{D}}_{t-1}^{*}\\ \;+\psi_{G^{\exp},t}F_{i,t-1}+e_{G^{\exp},t} \end{array}$$

(8) $$\begin{array}{l} {\hat{G}}_{t}^{rev}\;=\;\eta_{G^{rev},t}{\hat{G}}_{t-1}^{rev}+\lambda_{G^{rev}y,t}{\hat{y}}_{t-1}^{*}+\lambda_{G^{rev}b,t}{\hat{D}}_{t-1}^{*}\\ \;+\psi_{G^{rev},t}F_{i,t-1}+e_{G^{rev},t} \end{array}$$

$\psi_{G^{\exp},t}$, $\psi_{G^{rev},t}$ is the corresponding coefficient. $F_{i,t}={\left[f_{1,t},f_{2,t},\cdots \;,f_{n,t}\right]}^{\prime }$ is the control variable, *f*_1, *t*_, *f*_2, *t*_, ⋯*f*_*n, t*_ is the extracted common factor,the number of common factors is represented by n, and n is selected as 3 in this paper, we have also extracted and analyzed other numbers of common factors, and the results show that the number of factors has no significant impact on the core conclusions of this article. The specific robustness test results are kept for reference. The posterior mean trend of specific common factors will be introduced later. As can be seen from the theoretical analysis in the previous section, the key parameter is the degree of freedom of fiscal policy to respond to targeting $\lambda_{G^{\exp}y,t}$, $\lambda_{G^{\exp}b,t}$, $\lambda_{G^{\exp}y,t}$, $\lambda_{G^{rev}y,t}$, On the one hand, it reflects the relative preference of fiscal authorities to control actual government debt scale and the output gap, and on the other hand, it also reflects the internal feedback mechanism of fiscal policy to the macro economy. From the point of view of the adjustment mechanism, if $\lambda_{G^{\exp}y,t}<0$, it shows that the change of fiscal expenditure will be accompanied by the reverse change of the output gap, and the macro-economy tends to be stable. On the contrary, it shows that the fiscal expenditure and the output gap change in a forward direction, resulting in greater macroeconomic fluctuations. Similarly if $\lambda_{G^{\exp}b,t}<0$, it means that the fiscal authorities can effectively control the scale of the fiscal deficit through the application of fiscal rules. Contrary to the expenditure-based fiscal policy rules, in the income-based fiscal policy rule equation, when the adjustment parameters of the output gap and the scale of the fiscal deficit are positive numbers, it means that there is a good automatic stabilizer between the fiscal revenue and the output gap and the size of the fiscal deficit. Revenue-based fiscal policy rules have played a role in counter-cyclical regulation, which is conducive to stabilizing the scale of debt, which in turn is conducive to macroeconomic stability; on the contrary, it shows that the reverse changes in the scale of fiscal debt and tax adjustment have exacerbated the fluctuation of debt scale, which is not conducive to macroeconomic stability.

## Empirical Strategy

According to the theoretical analysis, fiscal policy tools are divided into expenditure type and revenue type, the policy targets are divided into output and government debt. Then the following regression equation is preliminarily constructed:


(9) $$\begin{array}{r} X_{t}=\varphi_{t}^{0}X_{t-1}+\varphi_{t}^{1}Y_{t-1}+\varphi_{2}^{t}F_{t-1}+\varphi_{t}^{2} \end{array}$$

$X_{t}={\left[{Ĝ}_{t}^{\exp},{Ĝ}_{t}^{\text{rev}}\right]}^{\prime }$including expenditure-based and revenue-based fiscal policy tools, $Y_{t-1}={\left[{ŷ}_{t-1}^{*},{\hat{D}}_{t-1}^{\text{*}}\right]}^{\prime }$ including fiscal policy pegging to target output and government debt. $\varphi_{t}^{0}$, $\varphi_{t}^{1}$ are (2 × 2) dimension coefficient matrices. *F*_*t*_ refers to the extracted common factor, which is equivalent to the control variable in the equation, $\varphi_{t}^{2}$ are (2 × *n*) dimension coefficient matrices, where *n* is the number of common factors. $\delta_{t}={\left[e_{G^{\exp},t},e_{G^{rev},t}\right]}^{\prime }$ and δ_*t*_ ~ *N*(0, Ω_2 × 2_), $\varphi_{t}^{0}$, $\varphi_{t}^{1}$, Ω are diagonal arrays. $\varphi_{t}^{0}=\left[\begin{array}{cc} \eta_{G^{\exp},t} & 0\\ 0 & \eta_{G^{rev},t} \end{array}\right]$, $\varphi_{t}^{1}=\left[\begin{array}{cc} \lambda_{G^{\exp},t} & 0\\ 0 & \lambda_{G^{rev},t} \end{array}\right]$, $\varphi_{t}^{2}=\left[\begin{array}{cccc} \psi_{1G^{\exp},t} & \psi_{2G^{\exp},t} & \cdots & \psi_{nG^{\exp},t}\\ \psi_{1G^{rev},t} & \psi_{2G^{rev},t} & \cdots & \psi_{nG^{rev},t} \end{array}\right]$. In the model parameter estimation part, refer to Primiceri (31), Negro and Primiceri (32) and Nakajima's (33) method for parameter estimation, split :


(10) $$\delta_{t}=A^{-1}\Sigma \zeta_{t}={\left[\begin{array}{cccc} 1 & 0 & \cdots & 0\\ a_{21} & \ddots & \ddots & \vdots \\ \vdots & \ddots & \ddots & 0\\ a_{k1} & \cdots & a_{k,k-1} & 1 \end{array}\right]}^{-1}\left[\begin{array}{cccc} \sigma_{1} & 0 & \cdots & 0\\ 0 & \ddots & \ddots & \vdots \\ \vdots & \ddots & \ddots & 0\\ 0 & \cdots & 0 & \sigma_{k} \end{array}\right]\xi_{t}$$

Where ξ_*t*_ ~ *N*(0, *I*_*k*_). Further order *A* = *tril*(α_*t*_, *k*), $h_{t}={\left[h_{1t},\ldots ,h_{kt}\right]}^{\prime }$, $h_{jt}=\log\sigma_{jt}^{2}$, *j* = 1, …, *k*. After this setting, the model has the form of stochastic volatility, which can verify the discretionary components of different economic periods.

The factor extraction equation is as follows:


(11) $$\begin{array}{r} W_{t}=\Gamma^{F}F_{t}+\Gamma^{V}V_{t}+\varepsilon_{t} \end{array}$$

*W*_*t*_ is (*N* × 1) dimensional information set, as far as possible to cover the macro variables involved in fiscal policy in China. *F*_*t*_ and the unobservable part and the observable part, respectively. Γ^*F*^ and Γ^*V*^ express the factor loading matrix of dimension and (*N* × *M*) dimension, *N*≫*K*+*M*, ε_*t*_ ~ *N*(0, Ω_*t*_).

The tendency of the posterior mean of the extracted common factors has apparent responses during periods of large-scale economic fluctuations in the Chinese economy (for example, during the global economic crisis, the period of the impact of the COVID-19, etc.), indicating that the extracted common factors can represent most of the information contained in the data set. Use the constructed model to deeply analyze the impact response of expenditure-type and revenue-type fiscal policies to various endogenous variables in the fiscal rule equation, and set the impulse response function as follows:


(12) $$\begin{array}{r} IRF_{P}\left(\lambda_{jt},\varpi_{t-1}\right)=E\left[P_{\text{t+}h}|\lambda_{jt},\varpi_{t-1}\right]-E\left[P_{t+h}|\varpi_{t-1}\right] \end{array}$$

Where *IRF*_*P*_ is the impulse response function of the variable *P*, λ_*jt*_ is unobservable impulse, ϖ_t-1_ is the historical information set for predicting P, recording all possible impulse response results. *h* represents the prediction step size and *E*[·] is the expectation operator.

## Data

In this section, we outline parameter estimation methods and descriptive statistics of related data. The variables involved in the model's central part include economic variables such as output gap, fiscal deficit, government expenditure, government revenue, etc. The selection and description of each data are as follows.

The implicit assumption in this article is that the output gap can be adjusted according to fiscal policy cycles and can include responses from automatic stabilizers' roles. Considering that this article's scope is limited to China, which is a closed economy, the net export value of goods and services is deducted from GDP (22). The nominal GDP is divided by the current quarter's CPI to get the real GDP (34), and X-12 seasonal adjustments are made to data with apparent seasonality. When calculating the output gap, considering that the commonly used HP filter method is too subjective when filtering the noise to obtain the long-term economic variables trend, this article chooses a more objective and rigorous wavelet filter. Obtain potential output (35). The output is divided into two parts: trend component and periodic component. The periodic component is a temporary disturbance and will not cause long-term effects on the output. The long-term trend component can be obtained by suppressing the periodic component through the wavelet filtering method:


(13) $$\begin{array}{r} y_{t}=y_{t}^{\text{*}}+\xi_{t} \end{array}$$

**Figure 1:**
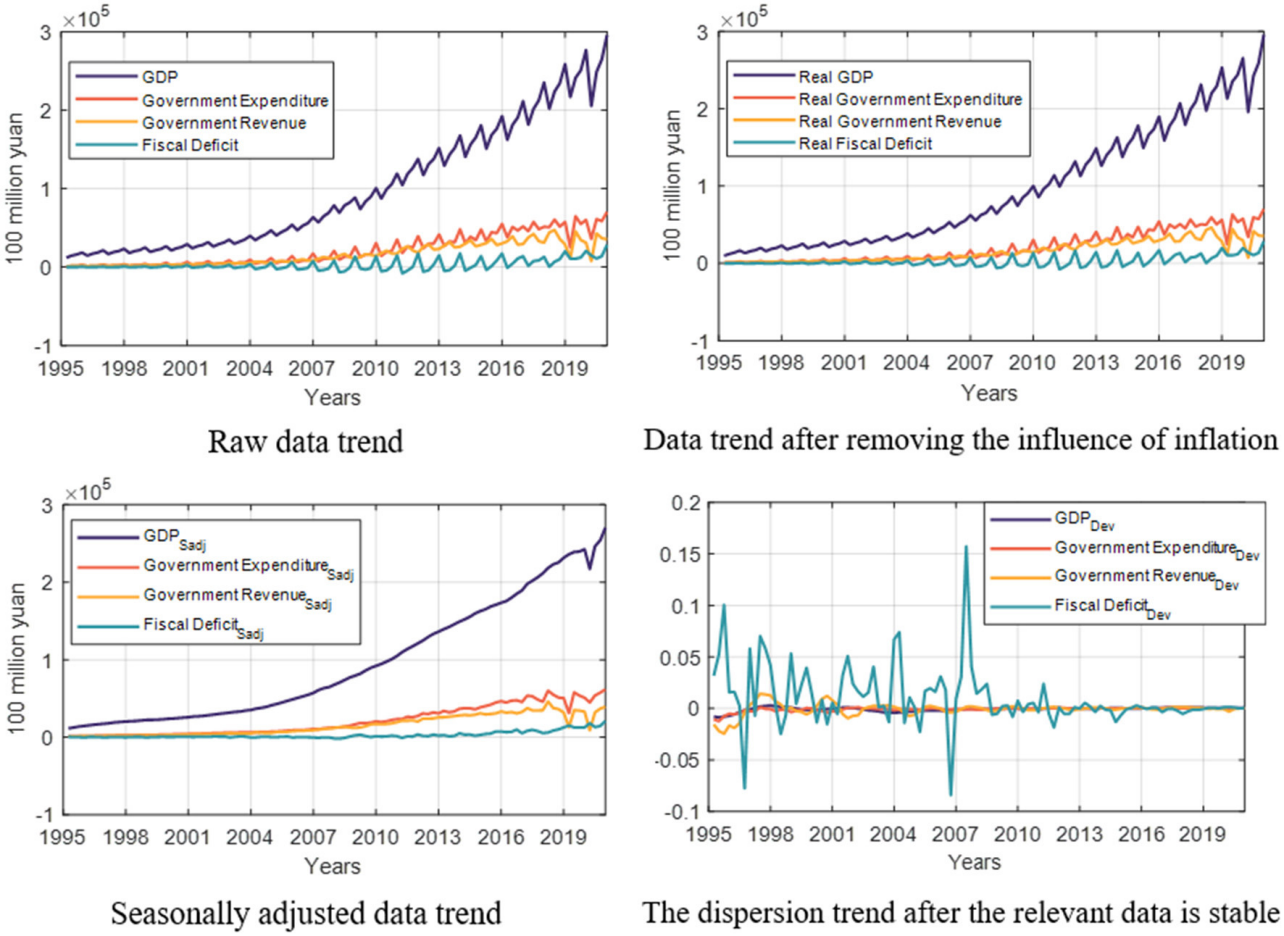
Related data trend description.

The trend part obtained through wavelet filtering $y_{t}^{\text{*}}$ is the potential output and is the output gap. The data processing process of fiscal expenditure and fiscal revenue is consistent with the output gap. The fiscal deficit is the fiscal revenue minus the state fiscal expenditure, and the dispersion ${\hat{D}}_{t}^{*}$ is calculated in the same way as above. The data trend chart is shown in Figure 1.

Considering the topic of this paper and the availability of data, a total of 86 economic variables, including China's actual economic activity level, currency level, and price level from the first quarter of 1995 to the fourth quarter of 2020 are selected for analysis. All data are quarterly and come from the wind database. Perform X12 seasonal adjustment on seasonal data and smooth uneven data through logarithmic or differential processing. Due to space limitations, specific data and common factor processing results are omitted in the text, and the processing process is kept for reference. Extract the unobservable common factors, as shown in Supplementary Figure 1.

## Results

**Figure 2:**
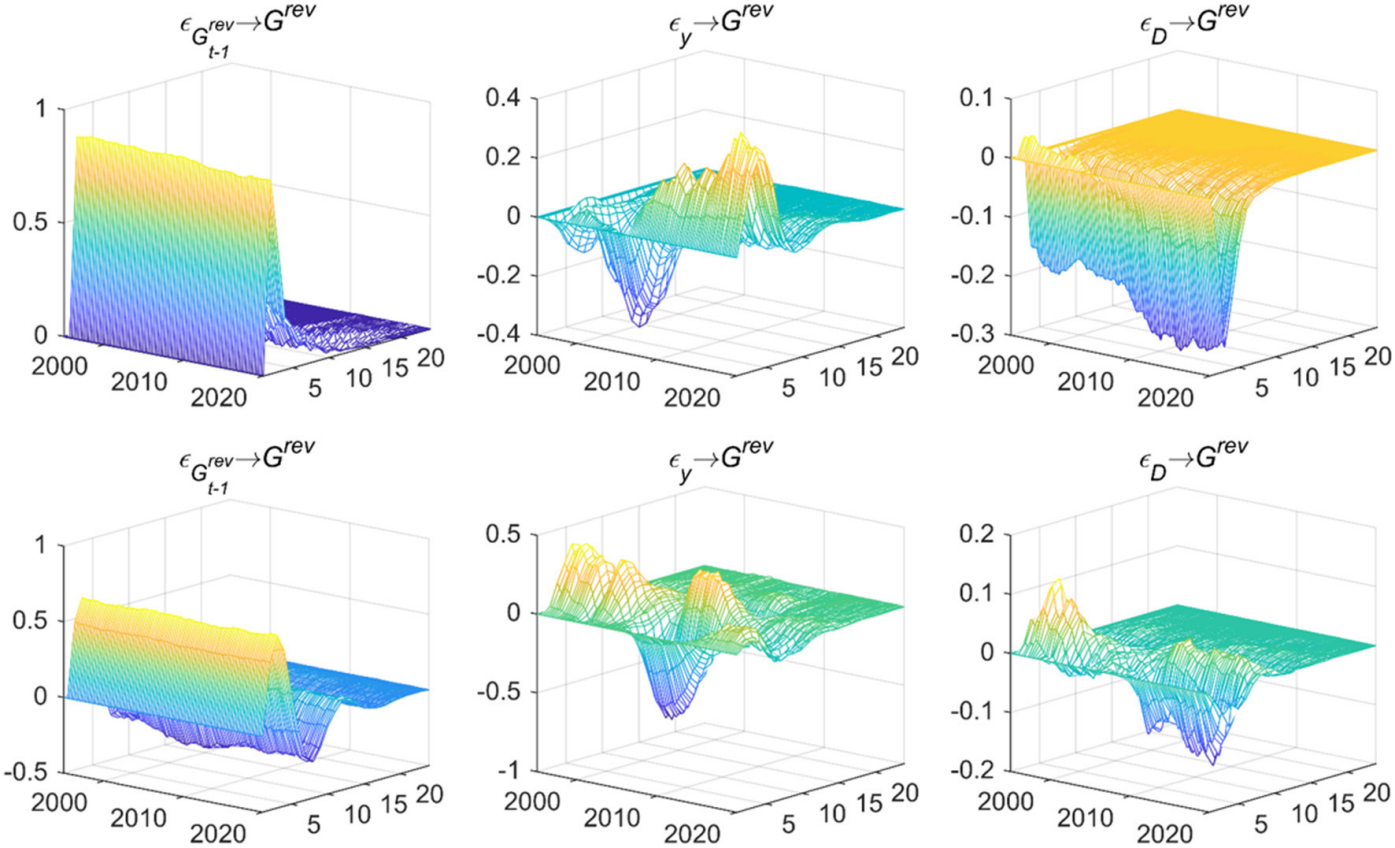
Dynamic feedback on fiscal policy rules. Each subfigure with the title of “*X*→*Y*” demonstrates the response of variable Y to an orthogonalized positive shock of variable X. In other words, X is an impulse variable, and Y is a response variable. One period in the figure denotes one season.

Figure 2 shows the empirical results, indicating both the expenditure-based and revenue-based fiscal policies are significantly affected by the previous period's policies. The dynamic feedback from output gap on fiscal expenditure ($\varepsilon_{y}\to G^{\exp}$) shows that before the global economic crisis, response value was negative and then turned positive. When the output gap is biased to the downside before the crisis begins, that is, the real output of the economy is lower than the potential output, fiscal expenditure increases, the contractionary fiscal deficit will prevent the further decline in real output, and the fiscal expenditure policy can play an effective role in counter-cyclical regulation, which is opposite to the process after the crisis. The feedback from output gap on fiscal revenue ($\varepsilon_{y}\to G^{\text{rev}}$) is similar to fiscal expenditure, which shows that during the crisis, the three-dimensional impulse response value fluctuates negatively, and the fiscal revenue policy is difficult to realize counter-cyclical regulation in this period, on the contrary, it will have a pro-cyclical impact on the economy. Such empirical results show that during the economic crisis, both fiscal revenue policy and fiscal expenditure policy are hard to achieve counter-cyclical regulation, so it is necessary to seek more supporting policy measures to promote macroeconomic growth in this special period. The effect of deficit scale on fiscal expenditure ($\varepsilon_{D}\to G^{exp}$) is negative during the sample period, which means the fiscal expenditure policy that focuses on deficit scale can play an effective role in counter-cyclical regulation. And the dynamic feedback from deficit scale on fiscal revenue ($\varepsilon_{D}\to G^{r\mathrm{ev}}$) shows that the impulse response before the global economic crisis is positive and the immediate feedback after the crisis is also positive, however, the lagging response is obviously negative fluctuations, indicating that the complex economic situation will weaken the positive feedback of fiscal revenue policy to regulate and control the deficit scale. The results suggest that both expenditure-based and revenue-based fiscal policies are challenging to play a countercyclical role during the economic crisis. In this particular period, it is necessary to turn to more supportive policy measures to promote macroeconomic growth.

**Figure 3:**
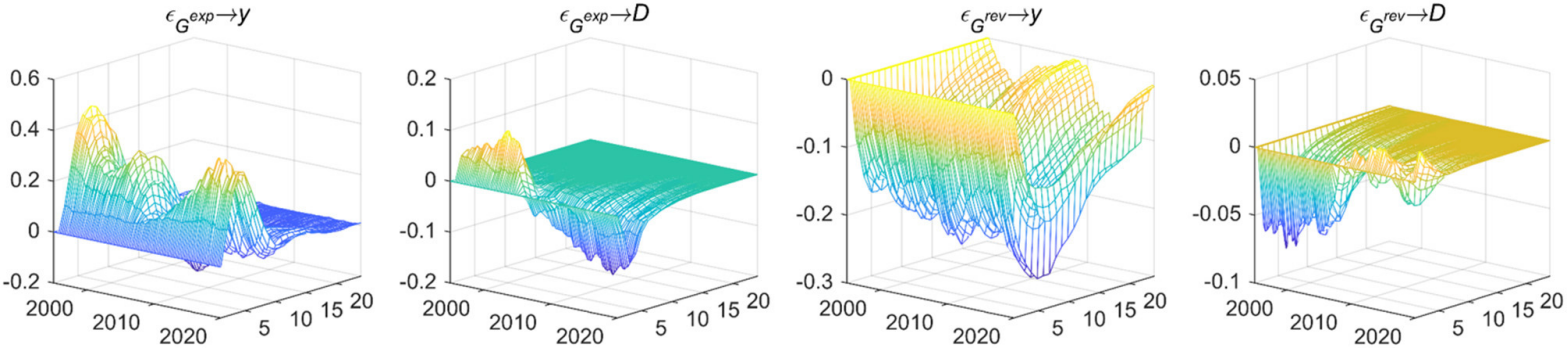
The macroeconomic control effect of fiscal policy. Each subfigure with the title of “*X*→*Y*” demonstrates the response of variable *Y* to an orthogonalized positive shock of variable *X*. In other words, *X* is an impulse variable, and *Y* is a response variable. One period in the figure denotes one season.

Fiscal expenditure shocks will lead to differential responses to output gaps and fiscal deficits in different periods as shown in Figure 3. Expenditure-based and revenue-based fiscal policies both have a time-varying effect on the output gap and the deficit scale. Before the crisis, they can effectively stabilize the output and deficit. However, maintaining the balance of government debt and boosting the economy faced unprecedented trade-offs after the crisis.

The above empirical results show that the external economic environment is an essential factor that affects the dynamic control effect of China's fiscal policy. Negative shocks in the economic system will have an impact on China's fiscal policy dynamic control mechanism. To deeply study the implementation characteristics of China's fiscal policy under COVID-19 epidemic, the Southeast Asian financial crisis (1997 Q3) and the global economic crisis (2008 Q3) are selected. China's economy has wholly entered a New Normal (2015 Q1) and the COVID-19's impact (2020 Q1). Several particular time points where the economy has suffered a greater negative impact will be further horizontally compared and analyzed in the sampling interval. Moreover, we observed that fiscal policy has become more discretionary during the COVID-19 outbreak, indicating that when the economy is hit by the COVID-19 pandemic, it tends to flexibly adjust fiscal spending and revenue policies according to the economic situation, which is consistent with the economic fact that China has introduced a series of fiscal support policies in response to the COVID-19 outbreak.

**Figure 4:**
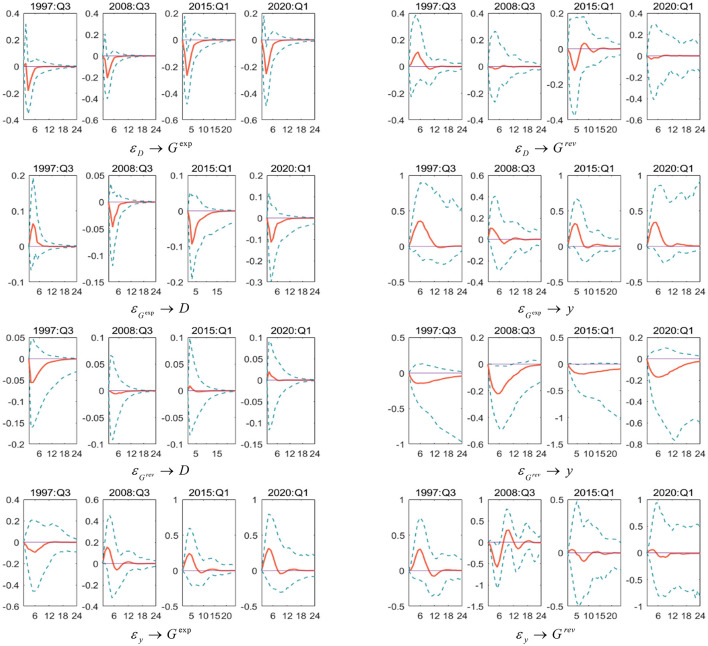
Dynamic feedback trajectory of fiscal policy rules in particular period. Each subfigure with the title of “*X*→*Y*” demonstrates the response of variable *Y* to an orthogonalized positive shock of variable X. In other words, *X* is an impulse variable, and *Y* is a response variable. One period in the figure denotes one season.

As shown in Figure 4, the following important conclusions can be drawn: first, when the economic system is facing negative shocks, the complex and fragile economic environment will increase the difficulty of controlling expenditure-based fiscal policies. Among them, the procyclical feedback between fiscal policy, output gap, and the scale of the deficit after the global economic crisis is rising. Secondly, as the process of fiscal policy control evolves, the interaction time with macroeconomic variables has shortened, indicating that China's fiscal policy control measures are more precise, the dynamic adjustment mechanism does not have a long-term effect even in a particular period. The impulse response results in a special period further confirm the conclusions of the particular test in the text. It is worth noting that the longer period of interaction between fiscal policy variables during COVID-19 epidemic means that the implementation of fiscal policy during COVID-19 epidemic should be particularly cautious, because inappropriate policies are likely to have long-term adverse effects on the economy.

## Conclusion

The main conclusions obtained in this paper are as follows:

From the empirical results, it can be seen that the time-varying parameter model can better capture the dynamic endogenous relationship between the output gap and the deficit's size during fiscal policy implementation, and there is evident continuity between expenditure-based and revenue-based fiscal policies. The fiscal expenditure policy has failed to show an excellent countercyclical control effect in recent years. Expansion of government fiscal expenditures during the economic downturn can quickly affect, but the government will spend a long time after fiscal expansion to calm economic fluctuations. The revenue-based fiscal policy's immediate regularity is apparent, effectively suppressing the output gap and reducing the deficit's size. It has a timely positive feedback endogenous adjustment effect, but the forward policy's regularity is significantly weakened. The dynamic fiscal policy control mechanism shows that since the global economic crisis, the impact of fiscal policy on the output gap and deficit scale has increased in procyclical effects, indicating that the complex and fragile economic environment has increased the difficulty of fiscal policy control, and the interaction time between fiscal policy variables has become significantly longer during the COVID-19 epidemic. Compared to targeting the deficit's size, fiscal expenditure and fiscal revenue have a more prominent feature of targeting the output gap. Also, due to article length and data availability limitation, the research conclusions still have limitations. In the future, it can be further developed and improved in terms of the mechanism of fiscal policy on macroeconomic regulation and the combination of macro and micro approaches.

## Data Availability Statement

The original contributions presented in the study are included in the article/Supplementary Material, further inquiries can be directed to the corresponding author/s.

## Author Contributions

SW: conceptualization, methodology, software, formal analysis, data curation, writing—original draft preparation, and writing—review and editing. BZ, SW, and HG: validation. BZ: investigation, resources, supervision, project administration, and funding acquisition. HG: visualization. All authors contributed to the article and approved the submitted version.

## Funding

This research was funded by the Key Project of National Social Science Foundation (Grant No. 20AZD43), National Education Science Planning Project, Ministry of Education Key Project Re-form and Evaluation of Compound Teaching Paradigm in Colleges and Universities Under the Smart Learning Environment (Grant No. DIA170365), and the National Natural Science Foundation of China (Grant No. 11901233).

## Conflict of Interest

The authors declare that the research was conducted in the absence of any commercial or financial relationships that could be construed as a potential conflict of interest.

## Publisher's Note

All claims expressed in this article are solely those of the authors and do not necessarily represent those of their affiliated organizations, or those of the publisher, the editors and the reviewers. Any product that may be evaluated in this article, or claim that may be made by its manufacturer, is not guaranteed or endorsed by the publisher.
