# Supplementary material for: Research on the Dynamic Feedback Mechanism of Fiscal Policy Regulation Under COVID-19: Evidence From China
Source: Front Public Health. 2022 Jul 5;10:931135. doi: 10.3389/fpubh.2022.931135 (PMC9295405; doi:10.3389/fpubh.2022.931135)
Supplement: Supplementary file 1 [file Data_Sheet_1.DOCX]

Supplementary Material

# Supplementary Figures

**Supplementary Figure 1.** Common factor posterior mean trend.
